# Insomnia and somnolence in idiopathic RBD: a prospective cohort study

**DOI:** 10.1038/s41531-017-0011-7

**Published:** 2017-03-20

**Authors:** Ronald B. Postuma, Jean-François Gagnon, Amelie Pelletier, Jacques Y. Montplaisir

**Affiliations:** 1 Department of Neurology, McGill University, Montreal General Hospital, Montreal, QC Canada; 2 Centre d’étude du sommeil et des rythmes biologiques, Hôpital du Sacré-Cœur de Montréal Montréal, 5400 Boul. Gouin Ouest, Montréal, H4J 1C5 QC Canada; 3 Department of Psychology, Université du Québec à Montréal, Montreal, QC Canada; 4 Department of Psychiatry, Université de Montréal, Montreal, QC Canada

## Abstract

Although some sleep disorders are markers of prodromal Parkinson’s disease and dementia with Lewy bodies, it is unclear whether insomnia and somnolence can predict disease. We assessed a large cohort of patients with idiopathic rapid eye movement sleep behavior disorder and age/sex matched controls, comparing the Epworth sleepiness scale, the Insomnia Severity Index, the Pittsburgh Sleep Quality Index, and polysomnographic variables. In those with repeated scales, we assessed change over time. Finally, we assessed whether sleep abnormalities predicted defined neurodegenerative disease. The 151 patients (age = 65.9, 75% male) completed sleep scales and were included. Epworth scores were similar between patients and controls (7.0+/−4.6 vs. 7.2+/−4.7, *p* = 0.77), and did not progress with time (change = +0.46+/−2.1, *p* = 0.45). Epworth scores were similar between those who developed neurodegenerative disease and those remaining disease-free (6.7+/−4.4 vs. 7.1+/−4.7, *p* = 0.70). Pittsburgh Index scores were higher in patients than controls (7.2+/−3.8 vs. 4.9+/−3.4, *p* = 0.004), mainly driven by the sleep disturbance/medication components (reflecting rapid eye movement sleep behavior disorder symptoms/treatment). Baseline Pittsburgh scores did not predict conversion to neurodegeneration, although sleep duration increased over time in those converting to neurodegenerative disease (+0.88+/−1.32 h, *p* = 0.014). Insomnia index scores were higher in patients than controls (10.0+/−5.5 vs. 6.35+/−4.66, *p* < 0.001), but declined over time (−1.43+/−5.09, *p* = 0.029) particularly in those converting to neurodegenerative disease. Finally, on polysomnogram, those with increased tonic rapid eye movement had higher risk of developing defined neurodegenerative disease (HR = 1.88, *p* = 0.039). In summary, we found that somnolence and insomnia do not predict neurodegeneration in idiopathic rapid eye movement sleep behavior disorder. As neurodegeneration progresses through prodromal stages, patients may have increasing sleep drive and duration.

## Sleep disorders: Predicting neurodegenerative disease

Neither daytime sleepiness nor insomnia predict neurodegenerative disease in patients with REM (rapid eye movement) sleep behavior disorder (RBD). Patients with RBD provide unique insights into the early symptoms of Parkinson’s disease (PD) as around 80% of them develop neurodegenerative disease. Previous studies in RBD patients have shown that loss of olfaction and abnormal color vision significantly increase the risk of developing neurodegenerative disease. To identify other predictive markers of neurodegeneration, Canadian researchers led by Jacques Y. Montplaisir assessed the sleeping patterns and behavior of 151 RBD patients over 11 years. Although patients reported more sleep disturbances than healthy controls, these changes did not predict whether they developed neurodegenerative disease. Interestingly, neither insomnia nor somnolence worsened over time in patients with RBD and small increases in sleep duration and reduced insomnia were reported by those that went on to develop neurodegenerative disease.

## Introduction

Daytime somnolence and insomnia are common features of neurodegenerative synucleinopathies. Insomnia, particularly sleep maintenance insomnia, occurs in up to 50% of Parkinson’s disease (PD) patients and is a common early feature of disease.^1^ Somnolence occurs in 30–40% of PD and 40–70% of dementia with Lewy bodies (DLB), and becomes more common as disease progresses.^2^ Although onset time varies, both sleep disorders can be seen at diagnosis, suggesting that they may be present in prodromal disease.

So far, however, evidence for somnolence and insomnia as prodromal markers is limited. Two general population studies reported that increased daytime sleep was associated with a 2–3 fold increased risk of developing PD in the future.^3, 4^ However, studies of early untreated PD have generally failed to find excessive daytime somnolence compared to controls.^5, 6^ For insomnia, a single study suggested a possible mild increased in PD risk (RR = 1.4) in those presenting to medical attention for insomnia in the prior two years,^7^ but no other studies have assessed whether insomnia predicts PD.

Idiopathic rapid eye movement (REM) sleep behavior disorder (RBD) is the strongest known predictor of PD and other synucleinopathies.^8^ Long-term studies suggest that over 80% of idiopathic RBD patients will eventually develop neurodegenerative synucleinopathy.^9–11^ Therefore, following patients with idiopathic RBD allows a unique opportunity to directly observe the prodromal stages of disease, and to test potential prodromal markers. RBD patients have been used to establish predictive value of many prodromal markers, including hyposmia, dopaminergic functional neuroimaging, impaired color vision, REM sleep atonia loss, autonomic symptoms, EEG slowing and quantitative motor testing.^12–18^ A single study of patients with idiopathic RBD found that Epworth sleepiness scale (ESS) scores were higher than healthy controls and predicted faster conversion to neurodegeneration in RBD.^19^ However, no other studies have assessed whether insomnia or somnolence predict outcome in idiopathic RBD.

Since 2004, we have been following a large cohort of idiopathic RBD^20^ patients. As part of clinical follow-up, patients completed sleep scales including the ESS, the insomnia severity index (ISI), and the Pittsburgh Sleep Quality index (PSQI). Therefore, in this study, we assessed whether insomnia and somnolence severity could predict development of defined neurodegenerative synucleinopathy in idiopathic RBD, and measured the evolution of these sleep disorders during prodromal stages in those who eventually developed neurodegenerative disease.

## Results

### Participant characteristics

In total, we evaluated 158 patients with idiopathic RBD. The 151 patients had at least one baseline sleep analysis and were included in this analysis. Mean age was 66.4+/−8.3, RBD duration from symptom onset was 8.7+/−9.3 years, and 75% were male. Mean age of controls was 68.9+/−8.5 and 74% were male.

Of the 151 included in this analysis, the 132 had at least one annual follow-up examination, of whom 50 developed neurodegenerative disease after a mean interval of 3.2+/−2.4 years from baseline evaluation (range = 1–11 years). The 26 patients had a primary diagnosis of dementia, of whom 24 had at least one cardinal manifestation of parkinsonism and 15 had full International Parkinson and Movement Disorders Society (MDS) parkinsonism criteria^21^ at disease diagnosis. Twenty four had a primary diagnosis of parkinsonism, of whom 20 had PD and three had multiple system atrophy.

### Epworth sleepiness scale

The 116 idiopathic RBD patients had a baseline ESS performed (Table 1). This was conducted on average 2.6+/−2.1 years before the most recent visit (for disease-free patients), or disease diagnosis (convertors). The mean ESS score was similar between idiopathic RBD patients (7.0+/−4.6) and controls (7.2+/−4.7, *n* = 57, *p* = 0.77). The proportion with abnormal ESS also did not differ (28.7% vs. 28.1%, *p* = 1.0)

**Table 1:** Baseline sleep scale scores

	Control	Idiopathic RBD	*p*	Disease-free	Converted	*p*
Epworth Sleepiness Scale	7.2+/−4.7 (*n* = 57)	7.0+/−4.6 (*n* = 116)	0.77	7.1+/−4.7 (*n* = 71)	6.7+/−4.4 (*n* = 33)	0.70
Abnormal Epworth	28.7%	28.1%	1.0	27.8%	33.3%	0.65
Insomnia Severity Index Total	6.4+/−4.7 (*n* = 54)	10.0+/−5.5 (*n* = 111)	<0.001	10.0+/−5.4 (*n* = 67)	10.4+/−5.9 (*n* = 63)	0.76
Abnormal ISI	24.5%	48.7%	0.004	50.8%	48.5%	1.0
Onset Insomnia	0.74+/−0.91	0.86+/−1.04	0.47	0.80+/−1.01	1.03+/−1.16	0.32
Maintenance Insomnia	2.11+/−1.39	2.66+/−1.67	0.036	2.71+/−1.59	2.64+/−1.78	0.83
Sleep Satisfaction	1.30+/−1.0	1.99+/−1.18	<0.001	2.0+/−1.19	2.14+/−1.21	0.59
Daytime Impact	0.94+/−0.90	1.51+/−1.21	0.003	1.57+/−1.26	1.45+/−1.12	0.64
QOL impact	0.56+/−0.75	1.30+/−1.10	<0.001	1.28+/−1.08	1.45+/−1.32	0.47
Worry about sleep	0.70+/−1.02	1.64+/−1.20	<0.001	1.63+/−1.15	1.76+/−1.32	0.63
Pittsburgh Sleep Quality Index Total	4.89+/−3.38 (*n* = 29)	7.18+/−3.80 (*n* = 111)	0.004	7.12+/−3.79	7.66+/−4.1	0.52
Abnormal PSQI	44.9%	68.5%	0.029	68.7%	70.6%	1.0
Bed Time	23:14+/−1:14	22:56+/−1:10	0.22	23:01+/−1:05	22:43+/−1:22	0.27
Wake Time	7:38+/−1:13	7:23+/−1:19	0.31	7:20+/−1:23	7:37+/−1:18	0.32
Sleep Duration (hours)	7.50+/−1.21	7.39+/−1.33	0.70	7.29+/−1.31	7.58+/−1.45	0.33
PSQI Subcomponents Sleep Latency	0.93+/−0.75	1.17+/−0.77	0.14	1.16+/−0.76	1.25+/−0.83	0.53
Sleep Quality	0.93+/−1.00	0.88+/−0.97	0.81	0.91+/−1.00	0.88+/−0.98	0.89
Sleep Duration	0.50+/−0.85	0.64+/−0.77	0.40	0.69+/−0.78	0.59+/−0.79	0.53
Sleep Inefficiency	0.40+/−0.85	0.56+/−0.90	0.39	0.54+/−0.88	0.72+/−1.02	0.38
Sleep Disturbance	1.21+/−0.56	1.49+/−0.65	0.036	1.48+/−0.64	1.53+/−0.71	0.72
Sleep Medications	0.28+/−0.80	1.51+/−1.44	<0.001	1.43+/−1.41	1.68+/−1.49	0.43
Daytime Dysfunction	0.66+/−0.72	0.92+/−0.78	0.11	0.91+/−0.73	1.00+/−0.89	0.61

*ISI* Insomnia Severity Index, *PSQI* Pittsburgh Sleep Quality Index

**Fig. 1:**
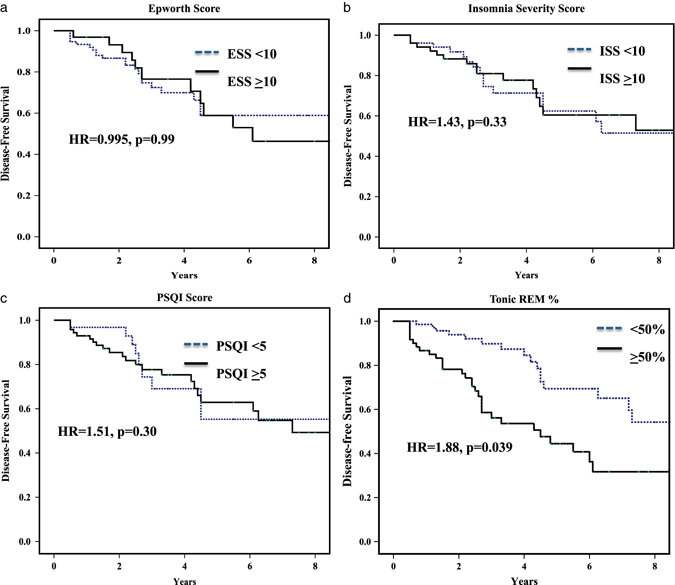
Shown is the Kaplan–Meier plot of disease-free survival of patients with idiopathic RBD, stratified according to the presence of sleep abnormalities: **a** Sleepiness, as assessed with the ESS, **b** insomnia as assessed with the Insomnia Sleep Index, **c** general sleep disturbance, as assessed with the PSQI, and **d** tonic REM, stratified to greater or less than 50% of epochs. The HR is for Cox regression analysis, adjusted for age and sex

104/116 patients had at least one prospective follow-up visit (Fig. 1, Table 1). We found no difference in baseline ESS scores between those who eventually converted and those still remaining disease-free (6.7+/−4.4 in convertors vs. 7.1+/−4.7 in disease-free, *p* = 0.70).

The ESS was repeated in 56 RBD patients at a mean interval (i.e. first to most recent score) of 2.3 years (Table 2). We found no change in ESS scores (average change = +0.07+/−3.1 points, *p* = 0.99). Moreover, there was no progression in ESS scores over time in those destined to develop disease (mean change = +0.46+/−2.1 points, *p* = 0.45).

**Table 2:** Change in Sleep Scale Scores over time

	Change: All idiopathic RBD	*p*	Change: converted	*p*
Epworth Sleepiness Scale	+0.07+/−2.1 (*n* = 56)	0.99	+0.46+/−2.1 (*n* = 13)	0.45
Insomnia Severity Index Total	−1.43+/−5.1 (*n* = 63)	0.030	−2.0+/−4.7 (*n* = 20)	0.072
Onset Insomnia	−0.064+/−1.10	0.65	−0.15+/−1.22	0.59
Maintenance Insomnia	−0.21+/−1.57	0.30	−0.45+/−1.05	0.083
Sleep Satisfaction	−0.33+/−1.35	0.056	−0.60+/−1.43	0.076
Daytime Impact	−0.17+/−1.06	0.22	−0.05+/−1.05	0.83
QOL impact	−0.17+/−1.1	0.21	−0.10+/−1.2	0.71
Worry about sleep	−0.48+/−1.29	0.004	−0.65+/−1.27	0.034
Pittsburgh Sleep Quality Index Total	−0.02+/−3.08 (*n* = 58)	0.97	−0.82+/−3.55 (*n* = 17)	0.36
Bed Time	−0:02+/−0:48	0.79	−0:06+/−0:43	0.58
Wake Time	+0:07+/−1:04	0.42	0:01+/−0:42	0.93
Sleep Duration (hours)	+0.24+/−1.34	0.18	+0.88+/−1.32	0.014
Subcomponents Sleep Latency	+0.12+/−1.30	0.48	−0.18+/−1.01	0.47
Sleep Quality	0.00+/−1.01	1.0	−0.059+/−1.39	0.86
Sleep Duration	−0.069+/−0.76	0.49	−0.44+/−0.66	0.014
Sleep Inefficiency	0.00+/−1.13	1.0	−0.44+/−0.81	0.040
Sleep Disturbance	−0.12+/−0.79	0.25	−0.24+/−0.75	0.21
Sleep Medications	−0.06+/−0.73	0.49	+0.35+/−1.37	0.31
Daytime Dysfunction	−0.017+/−3.08	0.53	−0.82+/−3.55	0.36

*RBD* REM sleep behavior Disorder

### Insomnia severity index

A total of 111 patients had assessment of their ISI, conducted an average of 2.6 years before last follow-up or disease diagnosis (Table 1). Overall, the mean ISI score in patients with idiopathic RBD was higher than controls (10.0+/−5.5 vs. 6.35+/−4.66, *n* = 54, *p* < 0.001). The 48.7% of RBD patients had abnormal ISI compared to 24.5% of controls (*p* = 0.004). When looking at specific items, differences were more marked for general sleep disturbances/worry/impact (i.e. questions 4–7) than for the direct insomnia questions. Nevertheless, there was a modest significant increase in sleep maintenance insomnia in RBD patients compared to controls (questions 2+3 = 2.7+/−1.7 vs. 2.1+/−1.4, *p* = 0.043).

Of the 111 patients with ISI, 100 had at least one follow-up examination (Fig. 1, Table 1). There was no difference in baseline ISI between those who developed disease vs. those who remained disease-free (10.4+/−5.9 vs. 10.0+/−5.4, *p* = 0.76). Neither was there any difference in the scores of any single ISI item.

The 63 patients had a repeated ISI, at a mean interval of 3.0 years between first and most recent score (Table 2). We observed a significant *decline* in ISI over time (mean change = −1.43+/−5.09, *p* = 0.029), moving towards control values with time. Although this declined both in those developing disease and those still disease-free, this was more clearly seen in those with disease (−2.00 in disease convertors vs. −1.16 in disease-free). When looking at subcomponents of the ISI in all RBD patients, the score reduction was primarily driven by questions related to sleep satisfaction/worrying about sleep. However, there was a borderline decline in sleep maintenance insomnia scores (questions 2 and 3) in those who developed disease (change = −0.45+/−1.05 points, *p* = 0.083).

### Pittsburgh sleep quality index

A total of 111 patients and 29 controls completed the PSQI (Table 1). The total PSQI was higher in patients than controls (7.2+/−3.8 vs. 4.9+/−3.4, *p* = 0.004), with the proportion of abnormal PSQI higher in RBD patients than controls (68.5% vs. 44.9%, *p* = 0.029). On subcomponent analysis, this difference was mainly driven by the ‘sleep disturbance’ (1.49+/−0.65, vs. 1.21+/−0.56, *p* = 0.035) and especially ‘sleep medications’ components (1.51+/−1.43 vs. 0.28+/−0.80). These subscales query symptoms of RBD itself and possible prodromal PD (see discussion). There was no clear difference in PSQI insomnia items; however, sleep maintenance insomnia is not directly queried in the PSQI.

Of the 111 patients, 101 patients had at least one prospective follow-up visit (Fig. 1, Table 1). We found no difference in baseline PSQI scores between those who converted and those who did not (7.1+/−3.8 in convertors vs. 7.7+/−4.1 in disease-free, *p* = 0.52). Similarly, there was no significant difference in any subcomponent between those who converted or remained disease-free.

A total of 58 patients had a follow-up PSQI, conducted a mean 2.8 years after the first PSQI (Table 2). For the entire group, there was no significant change in the total PSQI over time (change = −0.02+/−3.08 points), or in any subcomponent of the PSQI. When looking at those who converted to disease, there was no significant change in total PSQI (change = −0.82+/−3.6, *p* = 0.36). However, self-reported sleep duration increased significantly over time in those destined to develop disease (+0.88+/−1.32 h, *p* = 0.014). This change was significantly different from those who remained disease-free (disease-free = −0.02+/−1.26 h, *p* = 0.023). The increased duration was accompanied by a significant improvement in the PSQI sleep duration score (−0.44+/−0.66, *p* = 0.014) and better sleep efficiency (−0.44+/−0.81, *p* = 0.040), changes that were also significantly different from those patients who remained disease-free (sleep duration change in disease-free = 0.086+/−0.76, *p* = 0.012, efficiency change = +0.18+/−1.19, *p* = 0.026).

### Polysomnogram

We analzyed polysomnographic data for 151 patients and 85 controls (Table 3). There was no difference in sleep duration, sleep efficiency, or % of stages N1, N3, or REM sleep between patients and controls. A modest decrease in Stage 2 was seen in RBD patients (61.9+/−11.6 vs. 65.5+/−9.3, *p* = 0.015). As expected, there were large differences in REM tone measures between patients and controls.

**Table 3:** Polysomnographic variables

	Control (*n* = 85)	Idiopathic RBD (*n* = 151)	*p*	Disease-free	Converted	*p*
Sleep Latency (min)	19.1+/−24.4	23.7+/−24.8	0.17	21.8+/−22.7	28.4+/−30.4	0.18
Total Sleep Time (min)	383.2+/−63.4	384.3+/−61.7	0.90	389.5+/−57.5	373.8+/−71.6	0.19
Sleep efficiency (%)	79.6+/−12.3	81.8+/−10.6	0.15	82.7+/−9.5	79.8+/−12.3	0.15
Stage 1 %	12.2+/−6.8	13.1+/−9.2	0.43	13.1+/−8.4	13.8+/−11.4	0.71
Stage 2 %	65.5+/−9.3	61.9+/−11.6	0.015	60.9+/−11.0	63.2+/−12.6	0.29
Slow wave %	5.4+/−8.1	6.5+/−8.3	0.33	7.2+/−0.79	7.1+/−9.4	0.48
REM %	16.9+/−4.8	18.5+/−7.9	0.09	19.8+/−7.5	15.8+/−8.0	0.006
Phasic REM density %	11.5+/−7.9 (*n* = 59)	36.6+/−17.6	<0.001	35.5+/−17.0	34.7+/−18.0	0.81
Tonic REM %	7.9+/−9.0 (*n* = 59)	51.4+/−29.6	<0.001	46.1+/−30.4	58.4+/−27.0	0.019

Within RBD patients, comparing results according to disease outcome, there was no difference in total sleep time, sleep efficiency, or proportion of time spent in Stage 1, 2, or slow wave sleep between those who converted to disease vs. those who did not (Table 3, Fig. 1). Convertors had a modest decrease in % of sleep spent in REM sleep (15.8+/−8.0% vs. 19.8+/−7.5%, *pp* = 0.005). Also, patients who converted had higher tonic REM% (58.4+/−27.0% vs. 46.1+/−30.4%, *p* = 0.019), without any difference in phasic REM% (35.5+/−17.0% vs. 34.7+/−18.0%, *p* = 0.81). On Cox regression analysis adjusting for age and sex, having a tonic REM > 50% was associated with a hazard ratio (HR) of 1.88 for development of neurodegenerative disease (*p* = 0.039).

## Discussion

The key finding of this study was that although patients had mild increase in ISI and PSQI scores compared to controls, neither either excessive daytime somnolence nor insomnia predicted disease outcome in patients with idiopathic RBD. On prospective follow-up, we observed over time a subtle increase in sleep duration and decreasing complaints of insomnia specifically in patients destined to develop defined neurodegenerative disease.

With regards to daytime somnolence, we found a clearly negative result. ESS scores were similar in RBD as controls, did not increase over time, and did not predict outcome in idiopathic RBD. Note that our results are in contrast to a previous study on ESS in RBD, which found elevated ESS scores and a modest degree of predictive value, such that those with scores > 8 had a faster conversion time to neurodegenerative synucleinopathy.^19^ We cannot easily explain the discrepancy in results. There may be population and selection differences in the cohorts, or cohorts may answer questionnaires differently.

Results of the other two scales are less clear. At baseline, we found an elevation in both PSQI and ISI scores in idiopathic RBD patients compared to controls. However, the etiology of the increase is unclear. For the PSQI, the increase was mainly driven by the sleep disturbance and sleep medication components. Note that the sleep disturbance component contains questions possibly related to RBD itself (i.e. ‘bad dreams’), and several questions that may be confounded by other prodromal PD symptoms (nocturia, pain, and temperature disturbance).^22^ The sleep medication component would identify medications used as primary treatment of RBD (i.e. clonazepam, melatonin) and approximately 50% were taking one of these medications at baseline. The fact that there was no increase in the components of subjective sleep quality, sleep latency, duration, efficiency or daytime dysfunction suggests that primary insomnia and somnolence on the PSQI are not abnormal in idiopathic RBD. Similarly, on the ISI, most of the elevated scores were in the general sleep disturbance/satisfaction items, which may be related to symptoms of RBD itself. However, unlike the PSQI, the ISI specifically queries sleep maintenance insomnia. Here, we found a modest increase in idiopathic RBD patients. It is possible that some sleep maintenance problems can be directly due to RBD (i.e. wakening from agitated dreams). So, in summary, we did not find unequivocal evidence that insomnia per se is a prodromal feature of PD/DLB in RBD.

With regards to polysomnographic results, we confirmed our previous report that patients with more severe REM atonia loss were at higher risk of conversion to defined neurodegeneration.^14^ The fact that loss of REM atonia is progressive in RBD^23^ might suggest that patients with higher loss are more advanced in their disease, and therefore convert sooner (note, however, that follow-up duration did not differ between convertors and non-convertors). As in our previous publication, only tonic REM, but not phasic REM, was associated with disease conversion. It is unclear why this difference occurs. It is possible that there is a small subset of patients diagnosed with RBD mainly because of increased phasic REM, who have a form of RBD not related to underlying neurodegenerative synucleinopathy. Given the fact that the large majority of patients in our cohort converted to neurodegenerative disease, however, this proportion is likely quite small. On polysomnogram analysis, we also saw a slightly lower proportion of time spent in REM sleep in those who converted to defined neurodegenerative disease than those who remained disease-free. These differences were modest, and the etiology unclear, particularly given that control values were intermediate between the two groups. The possibility of a spurious finding needs to be considered. It is also possible that the more severe loss of tonic REM in convertors made definition of the REM Stage more difficult, thereby falsely reducing REM sleep scoring.

The assessment of evolution of sleep symptoms over time found some intriguing results. Overall, neither insomnia nor somnolence worsened over time in RBD. In contrast, insomnia scores reduced, and patients destined to develop disease reported increasing sleep duration over time. Some of the change in scales may be due to improvement in non-insomnia symptoms; for example, we have often informally noted spontaneous improvement in RBD symptoms as patients develop neurodegenerative disease. However, this should not explain the change in sleep duration. We do not think that this resulted from adding sedative medications. Of the 63 patients who had repeat ISI scores, 29 were taking clonazepam or melatonin at baseline, and 35 at follow-up; therefore, only 6/63 had new sedative medications. Average doses did not change over time in those taking clonazepam or melatonin (Clonazepam dose = 0.99+/−0.68 mg/day baseline and 1.11+/−0.88 mg last follow-up, Melatonin = 5.6+/−3.3 mg/day baseline and 6.1+/−3.5 mg at follow-up). Moreover, there was no correlation between change in ISI and change in these medications (Spearman correlation co-efficient = 0.07, *p* = 0.59). One might speculate that a general increase in sleep drive occurs as patients approach defined neurodegenerative disease, which remains below the threshold of excessive somnolence/involuntary daytime sleep. As neurodegeneration progresses (and perhaps with addition of symptomatic PD medications), this increased drive could progress to identifiable somnolence. Further studies are required to see if somnolence itself has a prodrome of a subthreshold increase in sleep drive.

Some limitations of the study should be noted. First, sleep questionnaires were gathered in an ad-hoc clinical fashion until 2013; therefore, many patients did not have a full questionnaire panel assessed. Nevertheless, we were able to obtain at least one completed questionnaire on 80% of our participants. Second, it should be noted that not all PD/DLB patients have RBD (RBD can be found in 30–50% of PD patients and > 75% of DLB patients).^24–26^ Because RBD marks a ‘diffuse-malignant’ subtype of PD with increased dementia and autonomic features,^27, 28^ and marks a subtype of DLB with decreased survival,^29^ our results cannot be reliably generalized to all PD/DLB patients. One might speculate that the RBD subtype may be more prone to somnolence rather than insomnia (given that somnolence commonly occurs in PD dementia and DLB), if so, population-based cohorts might be more likely to find sleep maintenance insomnia as a prodromal feature. Third, although the questionnaires are designed to assess specific syndromes, RBD itself can affect the way they are answered; for this reason, we took care to not simply report total scores at face value, but to look specifically within questionnaires for components that directly assessed insomnia or somnolence. Fourth, studies in idiopathic RBD may be confounded by floor effects (as we have observed in studies of autonomic dysfunction in RBD).^30, 31^ That is, if essentially all patients with RBD are in prodromal stages of neurodegeneration and the assessed marker becomes abnormal earlier in disease than RBD, the predictive value may not be observable. However, a floor effect is very unlikely here, given that scores were mostly similar between RBD patients and controls. Fifth, we assessed multiple outcomes in this study; the findings of increased sleep duration and reduced sleep maintenance insomnia in convertors were secondary variables, and should be considered as exploratory in nature.^32^ Finally, all our sleep measures are self-reported; self-reports of sleepiness in particular may be prone to underestimation by patients.^33^ It is possible that querying caregivers about somnolence would have given different results.

In conclusion, we did not find that somnolence or insomnia were prodromal markers of PD and DLB in idiopathic RBD. In RBD, there may be a subtle increase in sleep drive/duration that occurs as patients develop defined neurodegeneration.

## Methods

Details of the cohort, diagnostic criteria and annual follow-up protocol have been described in detail elsewhere.^20, 34–36^ The study was approved by the ethics board of the Sacre Coeur hospital, and all patients gave written informed consent to participate. Briefly, all patients had idiopathic RBD, as confirmed by polysomnography. All patients were free of parkinsonism and dementia at baseline. A comprehensive baseline evaluation by a neurologist (R.B.P.), and neuropsychological examination was performed, to examine markers of prodromal PD. Patients were then followed annually with the same protocol. On follow-up, diagnosis of parkinsonism was made according to UK brain bank and MDS criteria,^21, 37^ and the likeliest underlying diagnosis (i.e. PD or multiple system atrophy) was determined by a movement disorders specialist (R.B.P.). Dementia was diagnosed according to MDS criteria^38^ based on a consensus between the neurologist (R.B.P.) and the neuropsychologist (J.F.G.).

Age and sex-matched control subjects were selected from our previous cohort studies.^30^ All controls had polysomnography confirming the absence of RBD, and had the same baseline evaluation performed as idiopathic RBD patients. All were free of parkinsonism or dementia. Polysomnogram was performed according to standard protocol, with analysis of stages and quantification of REM atonia performed as described in detail elsewhere.^36^

Three sleep measures were assessed. The ISI is a 7-item questionnaire; the first three questions directly query sleep onset and sleep maintenance insomnia, whereas the latter four query sleep satisfaction and impact of sleep disorders on quality of life.^39^ A cutoff of 10 is considered as indicating insomnia. The PSQI is a 25-item inventory that queries habitual bed time and wake time (allowing assessment of circadian rhythm disturbance), with seven additional subcomponents: subjective sleep quality, sleep latency, sleep duration, sleep efficiency, various sources of sleep disturbance, sleep medication, and daytime dysfunction (somnolence and apathy).^40^ A cutoff score of five on the total index is used to define abnormal sleep. The ESS is a test for somnolence that queries the propensity to fall asleep in eight different situations.^41^ A cutoff of 10 is used to define pathologic sleepiness. We assessed these scales are part of the clinical assessment (until 2013, not all patients completed these scales). After 2013, sleep questionnaires were also assessed systematically as part of the research evaluation.

### Analysis

For comparison to controls, the first available baseline questionnaire (always in the idiopathic RBD state) was assessed in all patients. Total scores for each scale was used as the primary analysis; secondary analysis included scale subcomponents. To assess predictive value of sleep disorders, all patients who had baseline evaluation and at least one annual follow-up examination were included. The primary analysis was for the scores as continuous variables, using student t-test. Categorical variables (i.e. proportion abnormal) were assessed with Fischer Exact test. We also assessed predictive value of sleep variables using Cox regression also adjusting for age and sex. Finally, for all patients who filled out repeated questionnaires, we assessed change over time comparing the most recently-measured scores to baseline, using one-sample t-test (we analyzed polysomnogram variables at baseline only).
